# Exploring the oncogenic potential of circSOD2 in clear cell renal cell carcinoma: a novel positive feedback loop

**DOI:** 10.1186/s12967-024-05290-9

**Published:** 2024-06-27

**Authors:** Gao-sheng Yao, Liang-min Fu, Jun-shang Dai, Jin-wei Chen, Ke-zhi Liu, Hui Liang, Zhu Wang, Qiong Deng, Jie-yan Wang, Mei-yu Jin, Wei Chen, Yong Fang, Jun-hang Luo, Jia-zheng Cao, Jin-huan Wei

**Affiliations:** 1 Department of Urology, The First Affiliated Hospital of Sun Yat-Sen University, No. 58, ZhongShan 2nd Road, Guangzhou, 510080 Guangdong China; 2 Department of Obstetrics and Gynaecology, The Chinese University of Hong Kong, Hong Kong Special Administrative Region, China; 3 Department of Obstetrics and Gynecology, The First Affiliated Hospital of Sun Yat-Sen University, Guangzhou, Guangdong China; 4 Department of Urology, Affiliated Longhua People’s Hospital, Southern Medical University, Shenzhen, Guangdong China; 5 Institute of Precision Medicine, The First Affiliated Hospital of Sun Yat-Sen University, Guangzhou, Guangdong China; 6 Department of Urology, Affiliated Jiangmen Hospital of Sun Yat-Sen University, No.23 Haibang Street, Jiangmen, 529030 Guangdong China

**Keywords:** Clear cell renal cell carcinoma, Positive feedback axis, circSOD2, miR-532-3p, PAX5

## Abstract

**Background:**

Existing studies have found that circular RNAs (circRNAs) act as sponges for micro RNAs (miRNAs) to control downstream genes. However, the specific functionalities and mechanisms of circRNAs in human clear cell renal cell carcinoma (ccRCC) have yet to be thoroughly investigated.

**Methods:**

Patient cohorts from online databases were used to screen candidate circRNAs, while another cohort from our hospital was obtained for validation. CircSOD2 was identified as a potential oncogenic target, and its relevant characteristics were investigated during ccRCC progression through various assays. A positive feedback loop containing downstream miRNA and its target gene were identified using bioinformatics and validated by luciferase reporter assays, RNA pull-down, and high-throughput sequencing.

**Results:**

CircSOD2 expression was elevated in tumor samples and significantly correlated with overall survival (OS) and the tumor stage of ccRCC patients, which appeared in the enhanced proliferation, invasion, and migration of tumor cells. Through competitive binding to circSOD2, miR-532-3p can promote the expression of PAX5 and the progression of ccRCC, and such regulation can be salvaged by miR-532-3p inhibitor.

**Conclusion:**

A novel positive feedback loop, PAX5/circSOD2/miR-532-3p/PAX5 was identified in the study, indicating that the loop may play an important role in the diagnosis and prognostic prediction in ccRCC patients.

**Supplementary Information:**

The online version contains supplementary material available at 10.1186/s12967-024-05290-9.

## Introduction

The incidence of renal cell carcinoma (RCC) has been increasing for decades [1]. RCC accounts for approximately 3% of all malignant tumors [2] and is the second leading cause of death among urinary malignancies [1]. Clear cell renal cell carcinoma (ccRCC) is the predominant pathological type, representing approximately 70% of RCC cases worldwide [3]. Compared to other subtypes of RCC, ccRCC exhibits stronger oncogenicity and is often associated with a worse prognosis. However, the current therapeutic approaches for such malignant tumors, which have high rates of metastasis and recurrence, appear to be unsatisfactory [4, 5]. Therefore, further research is necessary to address the existing research gaps in our understanding of the molecular mechanisms and pathways that play a crucial role in the occurrence and development of ccRCC, aiming to provide improved patient management.

Circular RNAs (circRNAs) are a class of non-coding RNAs that are produced from exons in protein-coding genes. They are generated from precursor RNAs through back-splicing [6, 7]. One of their distinctive features is the presence of the covalently closed loops, with the absence of 5′ caps or 3′ poly-A tails [8, 9]. With the advancement and widespread use of high-throughput sequencing, a large number of circRNAs have been recently discovered in mammalian cells and are found to be widely present in the cytoplasm [8, 10, 11]. Previous studies have confirmed the critical role of circRNAs in the pathogenesis and progression of urological tumors [12–14]. In recent years, the mechanisms of circRNA function have gradually been revealed, and one widely accepted mechanism is the interaction between circRNAs and microRNAs (miRNAs), referred to as the miRNA sponge effect [10, 15]. However, the involvement of circRNAs and their functions in ccRCC remain unclear and require further elucidation.

In this study, we utilized our own dataset and open-access datasets, which include a circRNA microarray, to screen candidate circRNAs. Based on the screening criteria of strong correlation with ccRCC progression, circSOD2 (Arraystar ID: hsa_circRNA_004662, circBase ID: hsa_circ_0004662) was identified and verified in the FAH-SYSU patient cohort, showing higher expression in tumor samples than normal tissues. Previous studies have demonstrated the regulatory functions of circSOD2 in various diseases. Zhao et al. discovered that high expression of circSOD2 promotes cell proliferation in liver cancer and is associated with cancer advancement in vivo through the circSOD2/miR-502-5p/DNMT3a/JAK2/STAT3 signaling pathway [16]. Frey et al. identified the overexpression of circSOD2 in tumor (at least fourfold) in a cohort of 121 ccRCC and 91 normal controls. Interestingly, the expression level of circSOD2 in urine was found to be opposite to that in ccRCC tissues [17]. Although the reason for this discrepancy has not been elucidated, this finding suggests the potential of circSOD2 as a non-invasive molecular marker for diagnostic purpose. Furthermore, Mei et al. explored the involvement of circSOD2 in smooth muscle proliferation and neointima formation [18], while Li et al. investigated its function in osteoarthritis progression [19]. Additionally, in infected cells with virulent and avirulent M.tb strains, circSOD2 significantly expressed at a higher level in infected cells [20]. However, the role of circSOD2 in ccRCC remains unknown [21, 22]. In the subsequent experimental validation, we successfully demonstrated that circSOD2 can bind to miR-532-3p and inhibit its expression. The interaction further promots disease progression by upregulating the expression of the downstream gene PAX5. Remarkably, PAX5, in ture, can enhance circSOD2 expression, establishing a novel regulatory loop. Therefore, circSOD2 holds great potential as a promising biomarker and therapeutic target that could significantly improve the prognosis of ccRCC patients.

## Methods

### Bioinformatic analysis

Two datasets, comprising gene expression data and corresponding clinical data, were downloaded from the Gene Expression Omnibus (GEO, https://www.ncbi.nlm.nih.gov/gds/) to identify differentially expressed circRNAs in order to identify differentially expressed circRNAs in ccRCC. The first dataset, GSE100186, consists of four tumor samples along with their matched adjacent normal tissues. The second dataset, GSE137836, containing samples from three primary tumors and three metastatic tumors. Obtained from the Genomic Data Commons Data Portal (https://portal.gdc.cancer.gov), the TCGA cohort comprised 530 ccRCC samples and 72 normal control samples, in the form of RNA-seq files, as well as 516 ccRCC samples and 71 normal control samples of miRNA-seq files. None of the patients included in the TCGA cohort had undergone targeted therapy, immunotherapy, chemotherapy, or radiotherapy prior to surgical removal of the tumor. The clinical staging of ccRCC patients in the TCGA cohort was determined using the tumor staging system established by the American Joint Committee on Cancer (AJCC). Additionally, the pathologic grading of ccRCC patients was performed using the Fuhrman grading system.

To analyze the two GEO datasets, a robust multi-matrix averaging algorithm was employed. This algorithm effectively applied background correction and quartile normalization to each sequence, ensuring accurate and reliable results [23]. The expression level of the genes was determined by calculating the average value of multiple probes that corresponded to each gene, enabling a comprehensive assessment of gene expression. To identify differentially expressed genes, a threshold of |log2 fold-change|> 1 and *P* < 0.05 as set. Next, to narrow down the scope of screening, Venn diagram analysis was performed, and a higher threshold of |log2 fold-change|> 3 was adopted. Additionally, the values of datasets from the TCGA database were standardized as well, using Fragments Per Kilobase of exon model per million mapped (FPKM). For subsequent analysis, only patients with available expression profiles, clinicopathological information, and survival data were included. Both overall survival (OS) and disease-free survival (DFS) were used as prognostic endpoints. Kaplan–Meier method was employed to generate survival curves, with the median serving as the threshold to distinguish between high and low expression groups.

This research was conducted in accordance with the principles outlined in the Declaration of Helsinki. We complied with all database policies and obtained access to datasets from GEO and TCGA databases. For the data obtained from the GEO and TCGA databases, approval and informed consent from the institutional review board were not required, as these datasets were publicly available and de-identified.

### ccRCC patient samples

From the First Affiliated Hospital of Sun Yat-sen University (FAH-SYSU, Guangzhou, China), a total of 160 pairs of tissues diagnosed with ccRCC were acquired, along with the corresponding adjacent normal tissues. All patients included in the study underwent radical nephrectomy and did not have history of neo-adjuvant radiotherapy or chemotherapy. Detailed clinicopathologic features and follow-up records were accessible for each patient, with a median follow-up duration of 80 months. The SSIGN (Stage, Size, Grade, and Necrosis) scores were calculated for each patient, taking into account a variety of factors such as tumor stage, dimension, grade, and necrosis [24].

Tissue segments were promptly immersed in formalin upon collection from patients undergoing surgery, and were then embedded in paraffin for preservation. The remaining portions of these samples were rinsed with phosphate buffered saline (PBS) and stored at − 80 °C for subsequent analyses of RNA expression. Prior to their inclusion in the study, all patients were accurately informed, and informed consent was obtained from all participants. The Ethics Committee of the FAH-SYSU approved the collection and use of specimens for this study.

### Cell lines, authentication and culture conditions

The cell lines used in our study and cell culture protocols have been described previously [25]. In order to authenticate these cell lines, STR profiling was conducted at the time of purchase; the identities of these cell lines were confirmed and authenticated. Specifically, Human RCC cell lines 786-O, 769P, Caki-1, A498, ACHN and human renal proximal tubular epithelial cell line HK2 were purchased from American Type Culture Collection (ATCC). All cell lines involved in our study were cultured in a humidified atmosphere of 5% CO_2_ at 37 ℃. 786-O and 769P cells were cultured in RPMI-1640 (Invitrogen-Gibco), Caki-1 cells in McCoy’s 5A (Gibco) and A498, ACHN and HK2 cells in DMEM (Gibco). The culture medium for all kinds of cells contained 10% fetal bovine serum (Thermo Fisher Scientific) and 1% penicillin–streptomycin (Gibco). In actinomycin D assay, actinomycin D (Sigma-Aldrich) was added at a concentration of 2 μg/ml to the culture medium of 786-O and Caki-1 cells, and then incubated for 4, 8, 16, and 24 h.

The cell lines were consistently monitored to verify the absence of mycoplasma through initial confirmation by observation of cell growth status, rate, and medium traits under the microscope. For cells with suspected mycoplasma contamination, mycoplasma agarose gel electrophoresis will be used to detect the presence of contamination. Related reagents to be used were MycoEasy rapid mycoplasma detection kit (Cellapy), TAE (50 ×) (Beyotime), Agarose (Biofroxx), DNA ladder (Beyotime), and GoodView Nucleic Acid Dye (SBS Genetech). The supernatant of cell culture will be collected, and follow the Mycoplasma Detection Kit operation manual for subsequent operations The positive standard for mycoplasma is about 280 BP, and the primer dimer was about 80 BP [26].

### Treatments of nucleic acid: RNA extraction, use of RNase R, reverse transcription and quantitative real-time PCR (qRT-PCR)

The related procedures were performed as described previously [25]. The primer sequences are shown in Additional file 1 (Table S1).

### Transfection assays

To achieve stable transfection, short hairpin RNA (shRNA) targeting circSOD2 and a scrambled control were synthesized by GeneChem (Shanghai, China). In addition, GeneChem (Shanghai, China) synthesized human circSOD2 cDNA and inserted it into a GV689 vector to generate circSOD2 overexpression plasmids. An empty plasmid was used as the negative control. HEK293T cells were transfected with sh-circSOD2 and circSOD2 overexpression plasmids using Lipofectamine 3000 (Invitrogen). Lentivirus was collected from the supernatant 48 h later and subsequently used to infect ccRCC cells. After selection with puromycin, the efficiency of overexpression or knockdown was examined.

For transient transfection, RiboBio (Guangzhou, China) synthesized small interfering RNA (siRNA) targeting PAX5 along with a scrambled control. GenePharma (Suzhou, China) provided the miRNA mimics, miRNA inhibitors, and negative controls. Lipofectamine 3000 (Invitrogen, USA) was used as the transfection reagent for all cell lines. A list of the related sequences can be found in Additional file 1: Table S1.

### High-throughput sequencing of micro RNAs (miRNAs)

To investigate the potential downstream miRNAs of circSOD2, four ccRCC cell samples with circSOD2-sh and corresponding negative control samples were prepared. MiRNA sequencing was performed by Sangon (Shanghai, China). According to the structural characteristics of small RNA with 3′-end hydroxyl groups and 5′-end phosphoric acid groups, the samples in this project were processed by 3′-end connection, reverse transcription primer hybridization, 5′-end connector connection, cDNA single-strand synthesis, and library amplification, using the catalytic reaction characteristics of related enzymes and molecular biology techniques. After quality inspection and purification, libraries that met the Illumina platform sequencing criteria were obtained. RNA libraries were prepared using the standard Illumina sequencing protocol.

The information pretaining to this project has been stored in the GEO database and can be accessed using the GEO Series accession number GSE199053 (https://www.ncbi.nlm.nih.gov/geo/query/acc.cgi?acc=GSE199053).

### Fluorescence in situ hybridization (FISH)

Probes for circSOD2 labeled with Cy3 and miRNA-532-3p labeled with FAM were synthesized by GenePharma (Suzhou, China). Following the manufacturer’s instructions, the probes were hybridized with cells using a fluorescence in situ hybridization kit provided by GenePharma. To capture the images, a FV1000 confocal laser scanning microscope (Olympus, Japan) was utilized.

### Pull-down assay with biotin-coupled circSOD2

Biotinylated probes targeting the junction region of circSOD2 and control oligo-probes were designed by GenePharma (Suzhou, China). To construct probe-binding beads, the probes were incubated with streptavidin magnetic beads (Invitrogen, USA) for 2-h duration at room temperature. Afterwards, the magnetic beads covered with the probes were combined with the cell lysates and incubated at 4 °C overnight. The extracted miRNAs, which were pulled down, were then verified using qRT-PCR after treatment with TRIzol (Invitrogen, USA).

### Pull-down assay with biotin-coupled miRNA

GenePharma (Suzhou, China) synthesized biotinylated miR-532-3p mimics and a negative control miRNA, which were then transfected into the cells using Lipofectamine 3000 (Invitrogen, USA) for 48 h. Collected cell lysates were mixed with streptavidin magnetic beads and incubated at room temperature for a 2-h duration. Trizol was used to extract the pulled-down circSOD2 after washing the beads, followed by qRT-PCR to evaluate pull-down efficiency.

### Dual luciferase reporter assay

GeneCreate (Wuhan, China) synthesized and subcloned circSOD2 or 3’-UTR PAX5, which was either wild or mutant type, into pmirGLO. Additionally, the 3’-UTR of PADI1, MMP16, and HMGA2 were subcloned into the pmirGLO vector. HEK293T cells were inoculated into 96-well plates at a density of 3 × 10^3^/well, and co-transfected with pmirGLO-WT and pmirGLO-MUT vectors using miR-532-3p mimics or negative control. After 48 h, luciferase activity was measured using a Varioskan LUX machine (Thermo, USA) using a dual luciferase reporter kit (Promega, USA) and normalized to Renilla luciferase activity.

### Western blot

These procedures were performed as described previously [25]. The following antibodies were used in the study: PAX5 antibody (1:1000), PI3K antibody (1:1000), p-PI3K antibody (1:1000), AKT antibody (1:1000), p-AKT antibody (1:1000), mTOR antibody (1:1000), p-mTOR antibody (1:1000), and β-tubulin antibody (1:5000). All antibodies were purchased from Abcam (Cambridge, UK).

### Cell proliferation, migration and invasion assays

For the cell proliferation assay, 2 × 10^3^ cells were inoculated in 100 μL of culture medium in 96-well plates, and cell viability was measured using the Cell Counting Kit-8 (CCK-8) assay (Abbkine, China) at different times.

For Transwell migration and Matrigel invasion assays, the cells were starved in serum-free medium for 8 h beforehand. Then, approximately 1 × 10^6^ cells were suspended in 100 μL serum-free medium and added into the upper chamber (Corning, USA), which was coated without (in the migration assay) or with (in the invasion assay) 2% Matrigel (Corning, USA). The lower chamber was supplemented with a medium containing 10% FBS as the nutritional attractant. After incubation for a suitable time (8 h for the migration assay and 16 h for the invasion assay), the cells attached to the lower membrane surface were fixed with 4% paraformaldehyde and stained with 1% crystal violet. The cells on the surface of the upper membrane were then cleaned with cotton swabs, and five regions were randomly selected under an inverted microscope (Olympus, Japan) to calculate the number of migrated or invaded cells.

### Flow cytometry

Cells transfected with the different plasmids were cultured naturally for 48 h and digested with EDTA-free trypsin. The Annexin V Alexa Fluor 647/PI Apoptosis Detection Kit (4A Biotech, China) was used for double staining of all cells (including cells in the supernatant) according to the manufacturer's instructions. The proportion of apoptotic cells in each group was analyzed using a FACS flow cytometer (BD Biosciences, USA).

### Animal experiments in vivo

The Animal Care and Use Committee of Sun Yat-sen University approved all animal care and laboratory procedures, and all experimental procedures for mice in vivo adhered to ethical codes. The maximal tumor size was ensured not to exceed the maximum range. It was proved that males have higher infiltration of exhausted CD8 + T cells in renal cell carcinoma [27], in order to ensure tumorigenicity, we used male BALB/c nude mice (approximately four weeks old) purchased from Bestest (Zhuhai, China).

Male mice were randomly separated into two groups for the orthotopic xenograft tumor model. Approximately 1 × 10^6^ circSOD2-silenced 786-O-luc cells or control 786-O-luc cells were injected into the subcapsular interspace of the right kidneys after anesthesia with 1% pentobarbital sodium (50 mg/kg). Tumor size was observed using an in vivo imaging system at regular intervals. Following an 8-week period, the mice were euthanized and the transplanted tumor kidneys were measured in terms of weighed for subsequent analysis.

Male mice were randomly divided into two groups to establish a pulmonary metastasis model. Approximately 1 × 10^6^ circSOD2-silenced 786-O-luc cells or control 786-O-luc cells were administered via the tail vein. Tumor size was observed using an in vivo imaging system at regular intervals. Following After 8-week duration, the mice were euthanized, and their lungs were preserved with formalin. Subsequently, each lung mass was sectioned sequentially and pulmonary metastatic nodules were counted and recorded.

### Statistical analysis

R version 4.0.2 was utilized for bioinformatic analyses, and GraphPad Prism 8.0 was used for all statistical testing. Two-tailed Student’s t-test was used for intergroup comparisons. Chi-square test was used to analyze the association between the expression level of circSOD2 and clinicopathological parameters in ccRCC patients. Survival analysis was performed using the Kaplan–Meier method to assess patient survival, in combination with the log-rank test. Quantitative data derived from at least three repeat experiments were presented as means ± SD. Statistical significance was defined as *P* < 0.05.

## Results

### The filtration of circRNAs in RCC

**Fig. 1:**
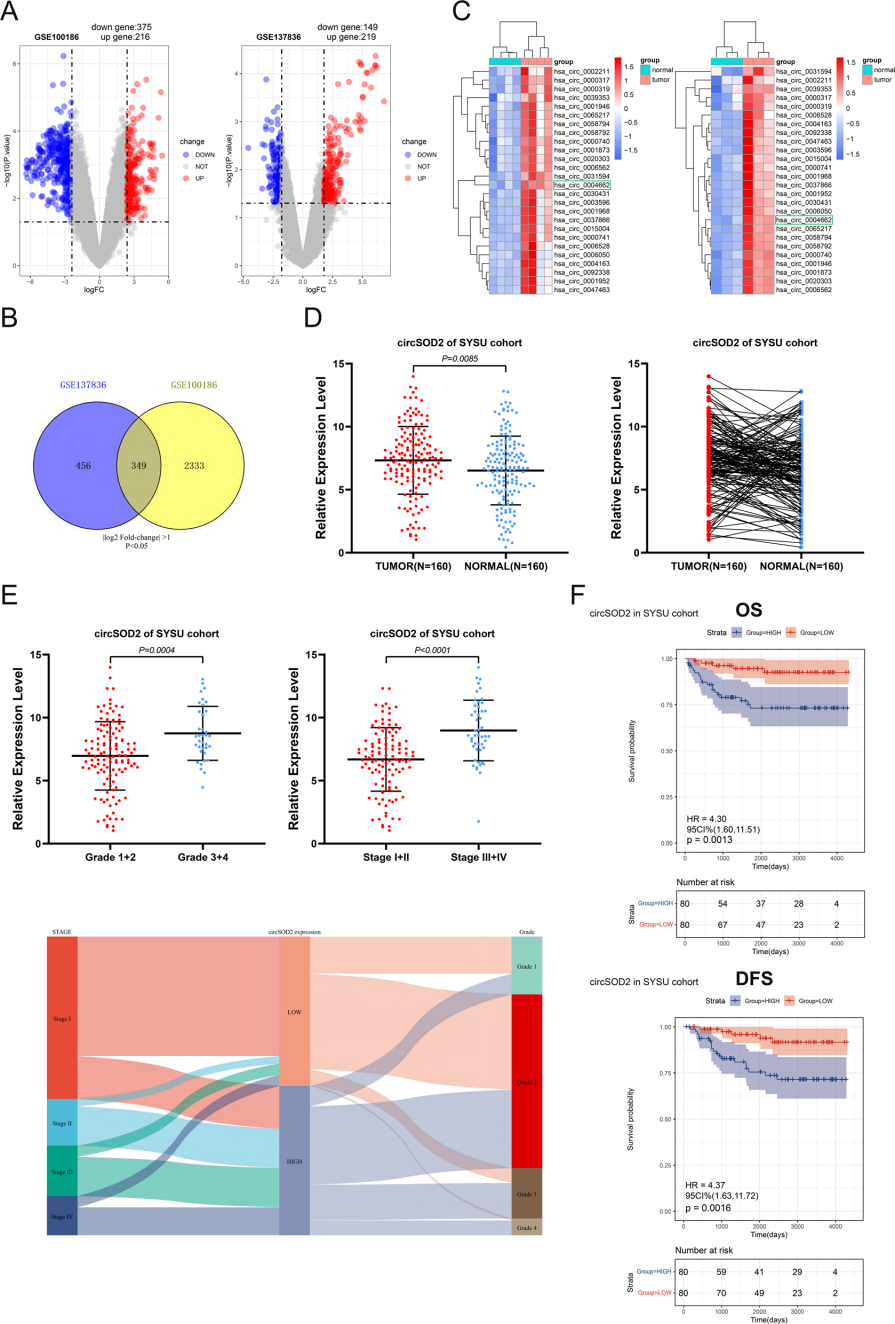
Identification of circSOD2 in ccRCC tissues. **A** Volcano plot of GSE100186 and GSE137836. Compared to the adjacent normal tissue (GSE100186) and the primary tumors (GSE137836), Red dots indicate significantly up-regulated circRNAs and blue dots indicate significantly down-regulated circRNAs. **B** Venn diagram of the intersection of differentially expressed circRNAs in two datasets. **C** The heat map shows the comparison of the top 26 genes with the most significant expression differences between the two circRNA microarrays. **D** In the SYSU-FAH patient cohort (160 pairs), circSOD2 expression was higher in tumor tissue, evaluated using qRT-PCR. **E** In the SYSU-FAH patient cohort, the more advanced the tumor grade and stage, the higher the circSOD2 expression level. **F** Kaplan–Meier analysis in the SYSU-FAH cohort demonstrated that patients with lower circSOD2 expression had a significantly better prognosis in DFS (the one at the top, *P* = 0.0013) and OS (the one at the bottom, *P* = 0.0016)

Two circRNA microarray expression profiles of RCC were downloaded from the GEO database for subsequent analysis: GSE137836 (consisting of three primary tumor tissues and their matched three metastatic tumor tissues) and GSE100186 (comprising four tumor tissues and their paired four adjacent normal tissues). By applying the cut-off criteria of |log_2_ fold-change |> 1 and *P* < 0.05, we identified 591 differentially expressed circRNAs (DEcircRNAs) in GSE100186, and 368 DEcircRNAs in GSE137836 (Fig. 1A). To narrow down the selection, we took the intersection of these two clusters, resulting in a subset of 349 candidate circRNAs (Fig. 1B) for downstream experiments. To further narrow down the intersection screening scope, the condition was restricted to |log_2_ fold-change |> 3, resulting in the identification of 26 conclusively DEcircRNAs (Additional file 2: Table S2). Two heatmaps were generated to visually represent the expression differences of these circRNAs in the two datasets (Fig. 1C).

### Association between CircSOD2 expression and prognosis of ccRCC patients

To validate the screening results obtained from bioinformatic analyses using clinical samples, qRT-PCR to assess the baseline expression level of these circRNAs in the FAH-SYSU patient cohort, consisting of 160 pairs was conducted. Compared to adjacent normal kidney tissues, a significant increase in circSOD2 expression in ccRCC tissues was observed (Fig. 1D). Then, the relationship between circSOD2 expression and clinicopathological features in the patient cohort was analyzed. Our finding indicated that higher expression level of circSOD2 is correlated with more advanced tumor stage and grade (Fig. 1E). Moreover, Kaplan–Meier analysis revealed that patients with lower circSOD2 expression exhibited a significantly better prognosis (Fig. 1F). Results from the chi-square test indicated a positive correlation between circSOD2 expression and the stage and grade of ccRCC, although no significant correlations with other clinical parameters (Table 1). Furthermore, after adjusting for clinical prognostic factors (age, TNM stage, and Fuhrman grade), the expression of circSOD2 remained an independent biomarker in the FAH-SYSU cohort. This suggested that higher circSOD2 expression may be associated with worse survival outcomes, including OS and DFS (Table 2).

**Table 1:** Correlations between circSOD2 expression levels and clinicopathological characteristics in 160 ccRCC patients

Parameters	Total (N = 160)	circSOD2 LOW(N = 80)	circSOD2 HIGH(N = 80)	P value	FDR
Age					
< 60 years	110 (68.75%)	57 (35.63%)	53 (33.13%)	0.61	1
≥ 60 years	50 (31.25%)	23 (14.38%)	27 (16.88%)		
Gender					
Female	50 (31.25%)	32 (20.00%)	18 (11.25%)	0.03	0.11
Male	110 (68.75%)	48 (30.00%)	62 (38.75%)		
Stage_T					
T1 + T2	123 (76.88%)	71 (44.38%)	52 (32.50%)	0.00074	0.0044
T3 + T4	37 (23.13%)	9 (5.63%)	28 (17.50%)		
Stage_N					
N0	154 (96.25%)	77 (48.13%)	77 (48.13%)	1	1
N1	6 (3.75%)	3 (1.88%)	3 (1.88%)		
Stage_M					
M0	148 (92.50%)	75 (46.88%)	73 (45.63%)	0.76	1
M1	12 (7.50%)	5 (3.13%)	7 (4.38%)		
TNM stage					
I + II	112 (70.00%)	68 (42.50%)	44 (27.50%)	0.000073	0.00051
III + IV	48 (30.00%)	12 (7.50%)	36 (22.50%)		
Fuhrman grade					
1 + 2	124 (77.50%)	71 (44.38%)	53 (33.13%)	0.0013	0.0064
3 + 4	36 (22.50%)	9 (5.63%)	27 (16.88%)		

P < 0.05 was considered to be statistically significant (Chi-square test)

**Table 2:** Univariate and multivariate Cox regression analyses of different parameters on overall survival (OS) and disease-free survival (DFS)

Parameters	OS				DFS			
	Univariate analysis		Multivariate analysis		Univariate analysis		Multivariate analysis	
	HR (95%CI)	*P* value	HR (95%CI)	*P* value	HR (95%CI)	*P* value	HR (95%CI)	*P* value
Age (≥ 60 years vs. < 60 years)	3.257 (1.456–7.286)	0.004	2.814 (1.214–6.526)	0.016	3.070 (1.374–6.859)	0.006	2.384 (1.036–5.486)	0.041
Gender (male vs. female)	2.123 (0.792–5.692)	0.135	1.682 (0.617–4.586)	0.309	1.972 (0.736–5.288)	0.177	1.785 (0.651–4.891)	0.260
TNM Stage (III + IV vs. I + II)	10.849 (4.273–27.549)	< 0.001	3.899 (1.299–11.706)	0.015	9.693 (3.816–24.619)	< 0.001	3.255 (1.071–9.894)	0.037
Fuhrman Grade (3 + 4 vs. 1 + 2)	5.265 (2.345–11.820)	< 0.001	2.175 (0.897–5.273)	0.085	5.354 (2.387–12.010)	< 0.001	2.383 (0.981–5.79)	0.055
CircSOD2 expression (high vs. low)	1.433 (1.209–1.697)	< 0.001	1.272 (1.055–1.534)	0.011	1.430 (1.212–1.688)	< 0.001	1.276 (1.065–1.529)	0.008

*OS* overall survival, *DFS* disease-free survival, *HR* hazard ratio, *CI* confidence interval

Receiver operating characteristic (ROC) analysis was conducted to assess the predictive accuracy of circSOD2 expression for OS in ccRCC patients. also showed that the expression level of circSOD2 can predict the OS of ccRCC patients with high accuracy, with higher accuracy when combined it with other clinical parameters. By analyzing the values of the area under the curve (AUC), it was found that AUC_circSOD2+SSIGN_ (= 0.898) was significantly higher than AUC_circSOD2_ (= 0.759) and AUC_SSIGN_ (= 0.828) (Additional file 3: Figure S1A,C). Similarly, curves in the DFS analysis of ccRCC patients also support this conclusion (Additional file 3: Figure S1B,D).

### Authentication and characteristics of circSOD2 in RCC

**Fig. 2:**
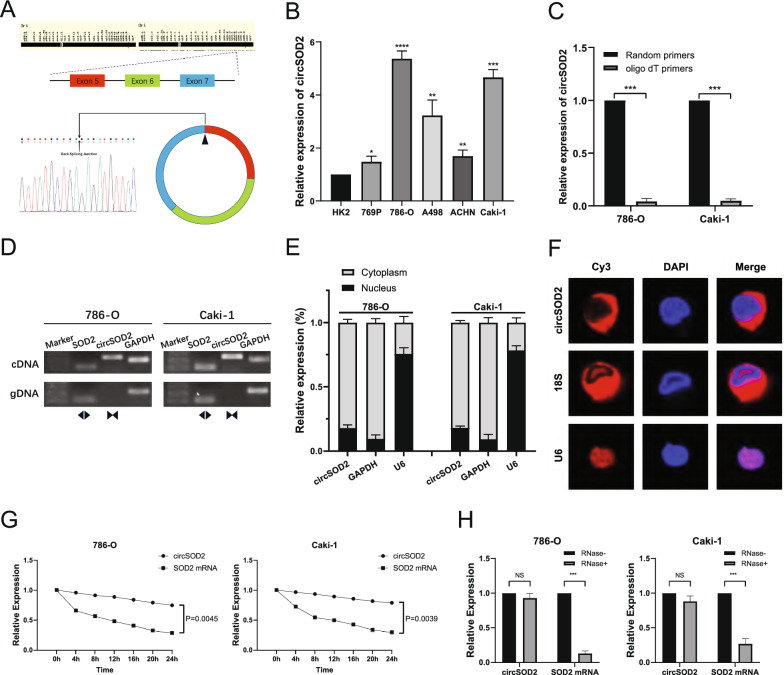
Authentication and characteristics of circSOD2 in RCC. **A** circSOD2 was verified by Sanger sequencing. The arrow indicates the back-splicing site of circ SOD2. CircSOD2 is derived by reverse splicing of exons 5, 6 and 7 of the SOD2 gene. **B** The expression of circSOD2 in RCC cells (769P, 786-O, A498, ACHN, and Caki-1) and human renal cortical proximal convoluted tubule epithelial cells (HK2). **C** qRT-PCR analysis of circSOD2 in the reverse transcription products using random primers or oligo dT primers. **D** The presence of circSOD2 was detected in 786-O and Caki-1 cells by qRT-PCR with convergent or divergent primers and validated by gel electrophoresis. GAPDH served as a control. **E** Comparison of circSOD2 expression in the nucleus and cytoplasm. **F** FISH analysis confirmed that circSOD2 was predominantly located in the cytoplasm. The nuclei were stained with DAPI. U6, 18S, and circSOD2 were labelled with Cy3. **G** Stability of circSOD2 and linear SOD2 was assessed by Actinomycin D treatment, followed by qRT-PCR at different time points. **H** Stability of circSOD2 and linear SOD2 was assessed by RNase treatment, followed by qRT-PCR. (NS, nonsignificant; *P < 0.05; **P < 0.01; ***P < 0.001; ****P < 0.0001)

CircSOD2 is the result of back-splicing of exons 5, 6, and 7, consisting of 462 base pairs, which were derived from the SOD2 gene locus on chromosome 6 (Fig. 2A). Sanger sequencing was performed to confirm the back-splicing junctions of circSOD2, and the results were consistent with those of circBase (Fig. 2A). To verify the expression level of circSOD2 in different cell lines, qRT-PCR was conducted, and the results confirmed that circSOD2 was significantly enriched in RCC cells (769P, 786-O, A498, ACHN, and Caki-1), compared to human renal cortical proximal convoluted tubule epithelial cells (HK2) (Fig. 2B). Random primers or oligo dT primers were used to detect reverse transcription products, and circSOD2 could hardly be detected by oligo dT primers, indicating that circSOD2 did not have a 3’ polyadenylated tail (Fig. 2C). To characterize the circular form of circSOD2, two types of primers were carefully designed: convergent and divergent primers. The results of PCR amplification with different primers and electrophoresis with the product showed that only cDNA with divergent primers could amplify circSOD2, while neither the convergent primers nor gDNA could amplify the desired product of PCR (Fig. 2D). Subcellular localization analysis (Fig. 2E) and FISH assays (Fig. 2F) further demonstrated that circSOD2 was mainly located in the cytoplasm of ccRCC cells. In addition, Actinomycin D and RNase R treatments were used to explore the stability of circSOD2, showing that circSOD2 has a longer half-life (Fig. 2G) and stronger resistance to degradation (Fig. 2H) than linear SOD2 mRNA.

### CircSOD2 promotes the progression of renal cell carcinoma in vitro and vivo

**Fig. 3:**
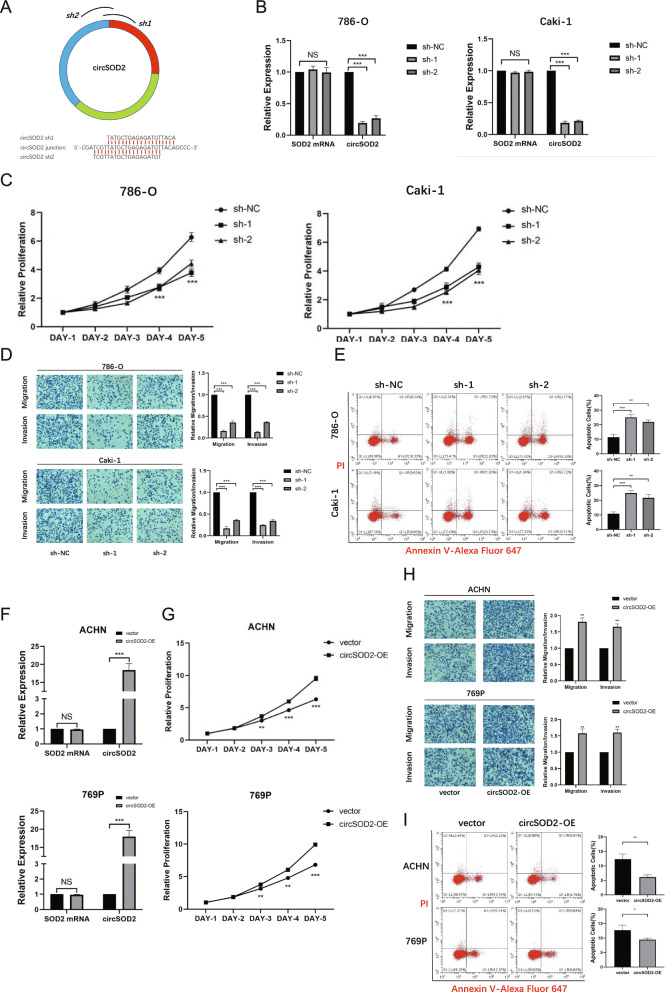
CircSOD2 promotes the progression of renal cell carcinoma in vitro. **A** Target sites of shRNAs used in the knockdown experiment. Both shRNAs target back-splice junction of circSOD2. **B** The circSOD2 knockout efficiency of 786-O and Caki-1 cells was measured by qRT-PCR. **C** CCK8 assays demonstrated that cell proliferation was inhibited after circSOD2 knockdown. **D** Transwell assays demonstrated that cell migratory and invasive capacity was inhibited after circSOD2 knockdown. **E** FACS assays demonstrated that cell apoptosis was enhanced after circSOD2 knockdown. **F** The circSOD2 over-expressed efficiency of 786-O and Caki-1 cells was measured by qRT-PCR. **G** CCK8 assays demonstrated that cell proliferation was enhanced after circSOD2 over-expressed. **H** Transwell assays demonstrated that cell migratory and invasive capacity was enhanced after circSOD2 over-expressed. **I** FACS assays demonstrated that cell apoptosis was inhibited after circSOD2 over-expressed. (NS, nonsignificant; *P < 0.05; **P < 0.01; ***P < 0.001; ****P < 0.0001)

To facilitate with exploring the biological function of circSOD2 and ro regulate the expression of circSOD2, shRNAs targeting the unique back-splicing junction region of circSOD2 were designed and constructed (Fig. 3A). After the introduction of shRNAs, the expression of circSOD2 was significantly knocked down in 786-O and Caki-1 cells, which contained relatively high levels of endogenous circSOD2, whereas the expression of linear SOD2 mRNA was not affected (Fig. 3B). Subsequently, the CCK-8 assay was performed to assess the proliferative capacity in 786-O and Caki-1 cells with circSOD2 downregulation, and was turned out that downregulation of circSOD2 would inhibit cell proliferation (Fig. 3C). Additionally, results of Transwell and Matrigel invasion assays indicated that decrease of circSOD2 can significant inhibit the migration and invasion capacities in 786-O and Caki-1 (Fig. 3D). Moreover, according to the flow cytometry results, downregulation of circSOD2 expression promoted apoptosis of 786-O and Caki-1 cells (Fig. 3E). In addition, a circSOD2-overexpressing lentivirus plasmid was synthesized to further study its function, and significant upregulation of circSOD2 was observed in ACHN and 769P cells, which had relatively low circSOD2 expression among the RCC cell lines (Fig. 3F). Consistent with the findings found in knockdown experiments, circSOD2-overexpressed cells showed increased proliferation (Fig. 3G), migration, and invasion (Fig. 3H) abilities, and decreased apoptosis levels (Fig. 3I). Comprehensively, these results suggested that circSOD2 potentially enhances the proliferation, migration and invasion of ccRCC cells.

**Fig. 4:**
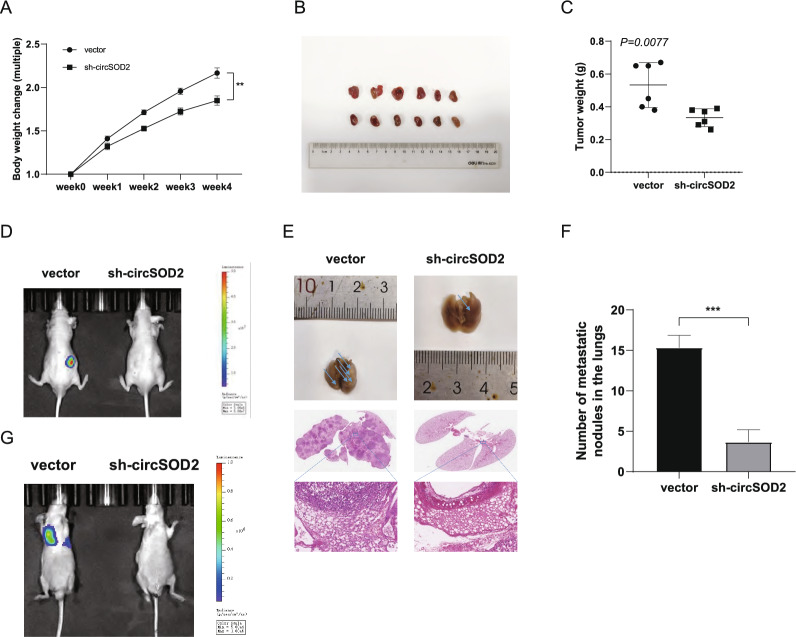
CircSOD2 promotes the progression of renal cell carcinoma in vivo. **A** Weekly mice body weight change in the orthotopic xenograft experiment. About 1 × 10^6^ circSOD2-silenced 786-O-luc cells or controlled 786-O-luc cells were injected into subcapsular interspace of the right kidneys. **B** The picture of the gross tumors in dissected from orthotopic xenograft model (right kidneys). **C** The mass of the gross tissue of the right kidneys. **D** Bioluminescence of the tumor tissue of kidney was detected by an in vivo bioluminescence imaging system. **E** Representative images of gross and microscopic HE stain of the tumor-infiltrated lung. **F** The metastatic foci in each mouse from two groups were counted under microscope and summarized. **G** Bioluminescence of the lung metastatic nodules was detected by an in vivo bioluminescence imaging system. (NS, nonsignificant; *P < 0.05; **P < 0.01; ***P < 0.001; ****P < 0.0001)

To confirm the tumorigenic effect of circSOD2 on tumor progression in vivo, BALB/c nude mice were used to develop an orthotopic xenograft tumor. In specific, 786-O cells with stable circSOD2 knockout or control were injected into the right renal subcapsule of mice in different groups. Based on the monitor of tumor growth status, we found that mice injected with circSOD2 knockdown cells showed a lower degree of cachexia than controls (Fig. 4A). Furthermore, four weeks after administering the injection, knockdown of circSOD2 significantly inhibited the growth of 786-O cells, which was reflected both in terms of volume and weight of the tumors, compared to those injected with control cells (Fig. 4B–D).

Next, to explore the metastatic potential of circSOD2 in vivo with a pulmonary metastasis model, 786-O cells were injected into male mice via the tail vein with stable circSOD2 knockdown or control vector. Four weeks after injection, indicated by in vivo imaging system and lung lesion measurement, the significant difference in numbers and volumes of metastatic nodules in the lungs had demonstrated that knockdown of circSOD2 inhibited the metastatic ability of 786-O cells in comparison with control cells. Previous studies had showed that more than 30% of RCC patients would develop metastatic tumor in lung [28–30]. Moreover, androgen receptor (AR) was demonstrated to be involved in RCC initiation and progression, leading to higher risk to lung metastasis [31–34]. Combined with the finding that circSOD2 can motivate the malignant advancement of non-small cell lung cancer [35], we decided to mainly focus on lung metastasis for the assessment of circSOD2’s capacity for distant metastasis. The mice were then sacrificed under anesthesia and their lungs were anatomized for further examination. Hematoxylin–eosin (H&E) staining showed a typical pattern of lung metastasis, and the number of metastatic nodules was significantly lower in the circSOD2 knockdown group than in the control group. This suggested that downregulation of circSOD2 inhibited lung metastasis of ccRCC in vivo (Fig. 4E–G).

### MiR-532-3p serves as specific targets of circSOD2 in ccRCC cells

**Fig. 5:**
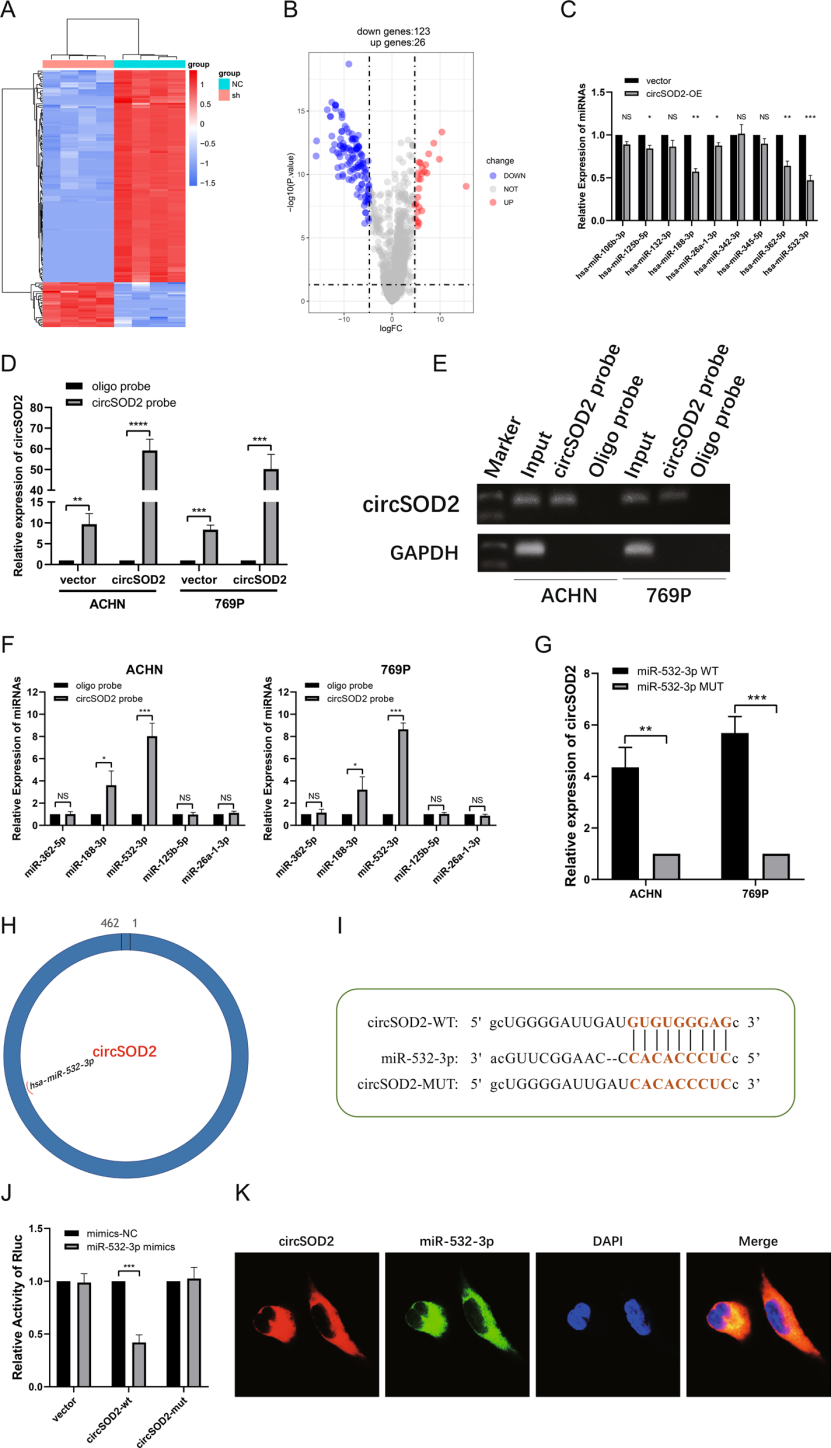
miR-532-3p is one of the targets of circSOD2 in ccRCC cells. **A, B** Heat map and volcano plot showed the difference of miRNA expression with or without circSOD2 knockdown. **C** The expression of candidate miRNAs were verified in circSOD2 overexpressed HK2 cells and negative control cells by qRT-PCR. **D, E** Relative expression detected by qRT-PCR and gel electrophoresis of circSOD2 in ACHN and 769P lysates after RNA pull down with circSDHC specific probe or oligo probe. Expression levels were normalized to oligo probe. GAPDH was used as negative control. **F** Relative levels of candidate miRNAs were detected by qRT-PCR after being pull down by circSOD2 probe or oligo probe. **G** Relative levels of circSOD2 in ACHN and 769P lysates captured by biotinylated wild-type miRNA-532-3p or mutant miRNA-532-3p. **H** Schematic diagram model showed the putative binding sites of miRNA-532-3p associated with circSOD2. **I** Schematic diagram of circSOD2 wild-type (wt) and mutant (mut) luciferase reporter vectors. **J** Luciferase reporter assay in HEK293T with vector, circSOD2 wild-type sequence and circSOD2 mutant sequence transfected with miR-532-3p mimics. Vector group was utilized as normalization control. **K** Cellular localization of circSOD2 (Cy3) and miRNA-532-3p (FAM) detected by FISH. Nuclear was label with DAPI dye. (NS, nonsignificant; *P < 0.05; **P < 0.01; ***P < 0.001; ****P < 0.0001)

It is widely known that circRNAs in the cytoplasm may play a part in miRNA inhibition as sponges [15]. Therefore, based on the subcellular localization of circSOD2, we attempted to identify the potential pathway of circSOD2 as a miRNA sponge. To identify the candidate downstream miRNA targets of circSOD2, the sh- and NC-samples of 786-O were used for high-throughput sequencing of miRNAs (refer to GSE199053 for specific results) (Fig. 5A, B). We strictly restricted the thresholds for screening to |log_2_ fold-change|> 5 and *P* < 1 × 10^–10^, and identified nine candidate miRNAs (Additional file 4: Table S3). Then, qRT-PCR was conducted to verify their expression patterns in treated HK2 cells, and five candidate miRNAs (hsa-miR-362-5p, hsa-miR-188-3p, hsa-miR-532-3p, hsa-miR-125b-5p, and hsa-miR-26a-1-3p) with significant differential expression were considered as potential targets of circSOD2 (Fig. 5C).

To demonstrate the direct interactions between circSOD2 and miRNAs in ccRCC cells, we constructed a biotin-labeled circSOD2 probe for the RNA pull-down assay. Compared with the control oligo probe, circSOD2 was significantly enriched using the circSOD2 probe in ACHN and 769P cells, which were transfected with the circSOD2 overexpression vector or negative control (Fig. 5D, E). Next, the probe was used to pull down the miRNAs that potentially bind to circSOD2 in both 786-O and Caki-1 ccRCC cells. The qRT-PCR results showed that, while both miR-532-3p and miR-188-3p upregulated significantly after RNA pull-down with circSOD2 probe, miR-532-3p was the most significantly clustered among the five candidate miRNAs, increased by over two times as much as miR-188-3p (Fig. 5F). Additionally, the binding capacity of miR-532-3p was analyzed as well by transfection. Biotin-labeled miR-532-3p and its mutants were stably transfected into ACHN and 769P cells to overexpress circSOD2, respectively, and circSOD2 captured by miR-532-3p was measured by qRT–PCR. Compared to the mutants, biotin-labeled miR-532-3p had a greater capacity to capture more circSOD2 (Fig. 5G). To perform the dual-luciferase reporter assay, wild-type (-wt) or mutant (-mut) circSOD2 sequences of the binding sites with miR-532-3p were inserted into the psiCHECK-2 vector (Fig. 5H, I). Next, the circSOD2-wt plasmid, circSOD2-mut plasmid, or empty plasmid and miR-532-3p mimic or negative control were co-transfected into HEK293T cells, followed by detection of Renilla luciferase activity. The results showed that compared with the negative control, Renilla luciferase activity was significantly decreased when circSOD2-wt and miR-532-3p mimics were co-transfected, supporting the hypothesis that miR-532-3p specifically binds to circSOD2 (Fig. 5J). Moreover, FISH analysis revealed that circSOD2 and miR-532-3p co-localized in the cytoplasm (Fig. 5K). In summary, the above experimental results collectively confirmed that circSOD2 can uniquely target miR-532-3p as a sponge to influence its expression.

### MiR-532-3p plays an inhibitory role in ccRCC by targeting PAX5

**Fig. 6:**
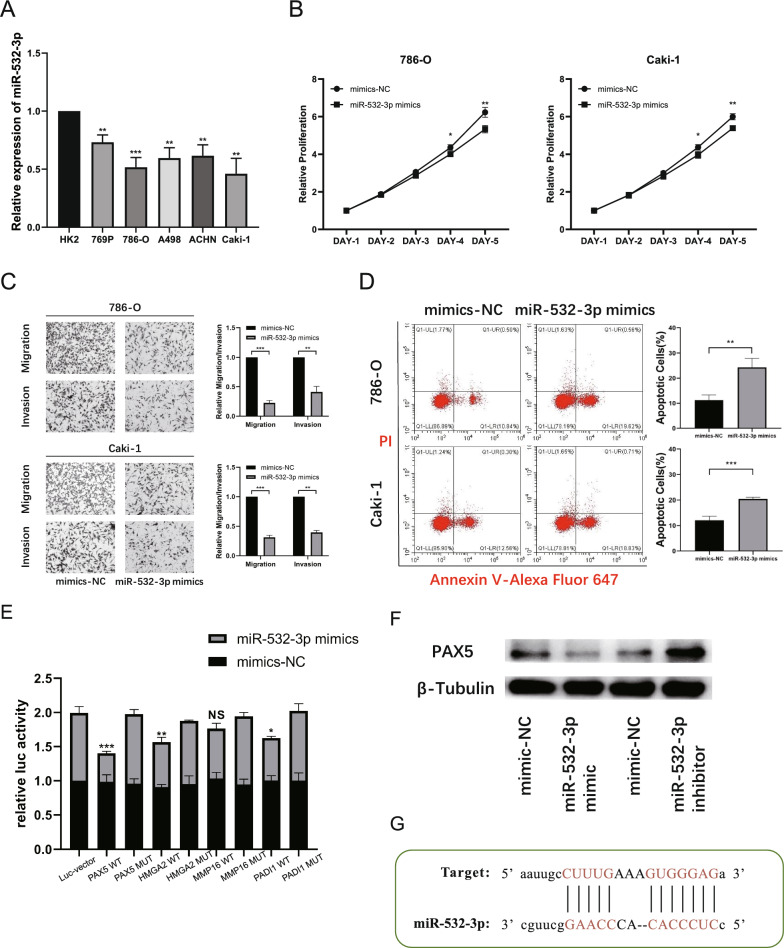
miR-532-3p plays an inhibitory role in ccRCC by targeting PAX5. **A** The expression of miR-532-3p in RCC cells (769P, 786-O, A498, ACHN, Caki-1) and human renal cortical proximal convoluted tubule epithelial cells (HK2). **B** CCK8 assays demonstrated that cell proliferation was inhibited after transfected with miR-532-3p mimics. **C** Transwell assays demonstrated that cell migratory and invasive capacity was inhibited after transfected with miR-532-3p mimics. **D** FACS assays demonstrated that cell apoptosis was enhanced after transfected with miR-532-3p mimics. **E** Luciferase reporter assay in HEK293T cells co-transfected mimics miR- miR-532-3p or mimics NC and candidate genes of Luc-wild-type or Luc-mutant. **F** Western blot analysis indicated that miR-532-3p could down-regulate PAX5 expression in RCC cells. **G** Binding site of miR-532-3p and PAX5. (NS, nonsignificant; *P < 0.05; **P < 0.01; ***P < 0.001; ****P < 0.0001)

We evaluated the role of miR-532-3p in ccRCC. Verification was conducted in various cell lines using qRT-PCR, and the expression of miR-532-3p was generally lower in ccRCC cells than in HK2 cells (Fig. 6A). To evaluate the specific function of miR-532-3p in ccRCC cells, we designed mimics and corresponding negative controls. The in vitro experiments demonstrated that the overexpression of miR-532-3p inhibited tumor proliferation, migration, and invasion, and enhanced cell apoptosis, which were observed in 786-O and Caki-1 cells (Fig. 6B–D).

Next, we assessed the expression patterns in different cohorts. In TCGA cohort, tumor tissues showed lower miR-532-3p expression than normal samples. In addition, from the survival data in the online database, patients with lower miR-532-3p expression had poorer OS and DFS (Additional file 5: Figure S2A). In addition, the differential expression profile of miR-532-3p in ccRCC tissues and normal tissues, as well as its association with patient prognosis (OS and DFS), were also similar to our analysis in the TCGA cohort (Additional file 5: Figure S2C). Similarly, in our FAH-SYSU patient cohort, the expression level of miR-532-3p was negatively correlated with circSOD2, which was confirmed by the qRT-PCR results (Additional file 5: Figure S2E).

Subsequently, TargetScan [36] (http://www.targetscan.org/vert_80/), miRDB [37] (http://www.mirdb.org/), and miRmap [38] (http://mirmap.ezlab.org) were used to predict the target genes of miR-532-3p, and four candidate genes (PAX5, HMGA2, MMP16, and PADI1) were identified. Further screening using a double-luciferase reporter gene assay showed that the vector containing the PAX5-wt sequence significantly reduced luciferase activity, whereas the mutant sequences showed no difference (Fig. 6E). Western blotting also confirmed the correlation between miR-532-3p and PAX5. The expression of PAX5 was significantly decreased after transfection with miR-532-3p mimics, whereas it was upregulated after transfection with the miR-532-3p inhibitor (Fig. 6F). Therefore, we concluded that miR-532-3p could downregulate PAX5 expression in RCC cells, and according to the TargetScan database, its binding type was summarized as a classical 8mer site (Fig. 6G).

**Fig. 7:**
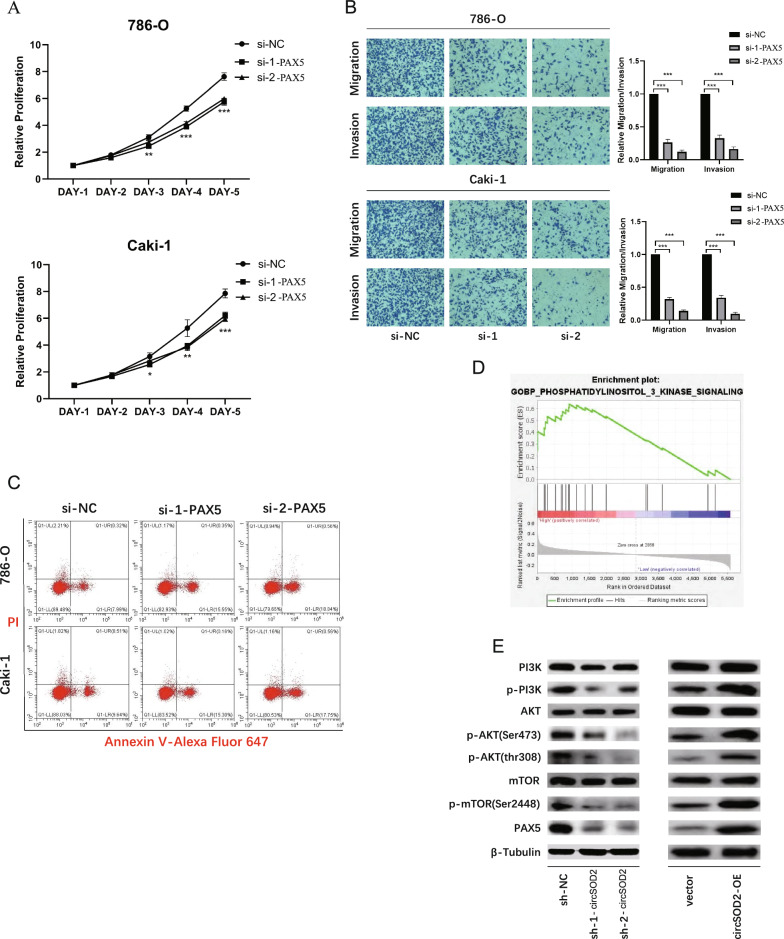
Down-regulation of PAX5 suppresses proliferation, migration and invasion of RCC cells in vitro. **A** CCK8 assays demonstrated that cell proliferation was inhibited after PAX5 knockdown. **B** Transwell assays demonstrated that cell migratory and invasive capacity was inhibited after PAX5 knockdown. **C** FACS assays demonstrated that cell apoptosis was enhanced after PAX5 knockdown. **D** GSEA analysis in TCGA patients revealed that PAX5 may be involved in PI3K signaling. **E** Western blot analysis indicated that circSOD2 promotes the ccRCC progression through PI3K-AKT-mTOR pathway. (**A–C** si-NC, referred to a scramble control of siRNA; si-1 & si-2, referred to siRNA targeting to PAX5 with different sequences; **E** sh-NC, referred to a scramble control of shRNA; sh-1 & sh-2, referred to shRNA targeting to circSOD2 with different sequences). (NS, nonsignificant; *P < 0.05; **P < 0.01; ***P < 0.001; ****P < 0.0001.)

PAX5, the gene encoding paired box 5, is specifically upregulated in many cancers, including diffuse large B-cell lymphoma [39], breast cancer [40], glioblastoma [41], and pancreatic cancer [42]. However, the regulatory mechanism of PAX5 in ccRCC has not been explored. In subsequent cell functional experiments, the downregulation of PAX5 inhibited the proliferation (Fig. 7A), migration, and invasion (Fig. 7B) of ccRCC cells and promoted apoptosis (Fig. 7C), with the use of siRNA targeting PAX5. Then we intended to set our insights into the patient cohorts. Analysis of the FAH-SYSU cohort revealed that the expression level of PAX5 was higher in tumor tissues and, to some extent, could predict OS and DFS in patients with ccRCC (Additional file 5: Figure S2D). Moreover, the negative correlated expression pattern of miR-532-3p and PAX5 were demonstrated in the FAH-SYSU cohort (Additional File 5: Figure S2F). A comparable profile was observed in TCGA cohort (Additional file 5: Figure S2B, G). Taken together, these results suggested that PAX5 can be regarded as a target gene of miR-532-3p, acting as an oncogene in ccRCC cells.

GSEA (https://www.gsea-msigdb.org/gsea/index.jsp) [43] was used to predict the downstream mechanism of PAX5, and the results showed that the PI3K pathway may be one of the most relevant signaling pathways (Fig. 7D), which is consistent with a previous study [44]. Western blot analysis indicated that the phosphorylation of PI3K, Akt, and mTOR was decreased in cells with circSOD2 knockdown, using shRNA to achieve a more stable transfection, while circSOD2-overexpressed cells showed the opposite trend (Fig. 7E).

### CircSOD2 rescues the tumor suppressive effect of miR-532-3p

**Fig. 8:**
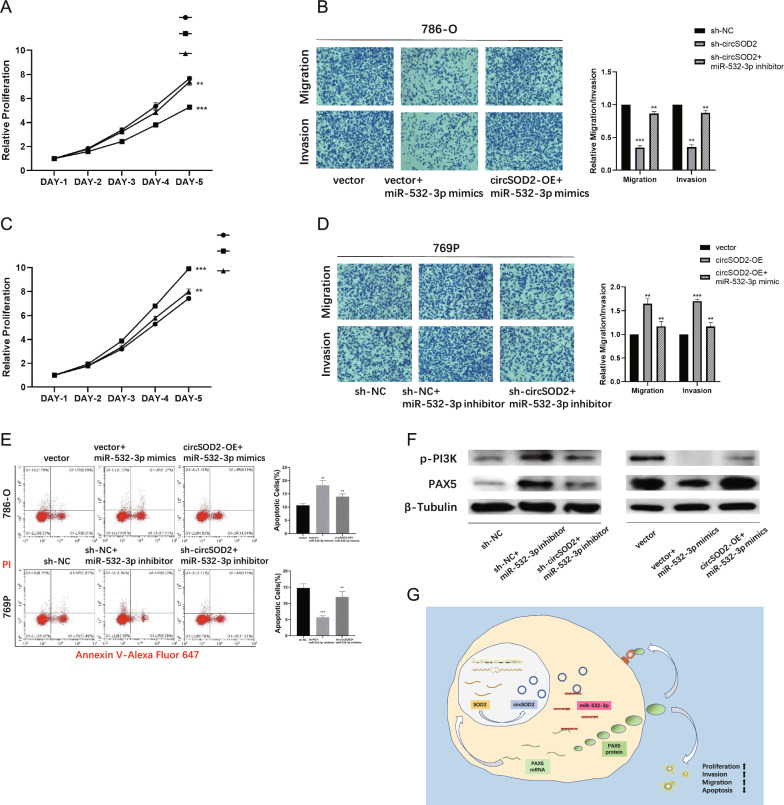
circSOD2 rescues the tumor suppressive effect of miR-532-3p. **A** Cell proliferation ability of 786-O transfected with control vector, miR-532-3p mimics alone or circSOD2 overexpressed plus miR-532-3p mimics. **B** Cell migration and invasion abilities of 786-O transfected with control vector, miR-532-3p mimics alone or circSOD2 overexpressed plus miR-532-3p mimics. **C** Cell proliferation ability of 769p transfected with control vector, miR-532-3p inhibitor alone or circSOD2 shRNA plus miR-532-3p inhibitor. **D** Cell migration and invasion abilities of 769p transfected with control vector, miR-532-3p inhibitor alone or circSOD2 shRNA plus miR-532-3p inhibitor.** E** Apoptosis of 786-O transfected with control vector, miR-532-3p mimics alone or circSOD2 overexpressed plus miR-532-3p mimics (above), and apoptosis of 769p transfected with control vector, miR-532-3p inhibitor alone or circSOD2 shRNA plus miR-532-3p inhibitor (below). **F** Western blot of PAX5 and p-PI3K levels after 769p transfected with control vector, miR-532-3p inhibitor alone or circSOD2 shRNA plus miR-532-3p inhibitor (left), and 786-O transfected with control vector, miR-532-3p mimics alone or circSOD2 overexpressed plus miR-532-3p mimics (right). **G** The diagram of the signal pathway. (NS, nonsignificant; *P < 0.05; **P < 0.01; ***P < 0.001; ****P < 0.0001)

To investigate whether circSOD2 promoted RCC progression by sponging miR-532-3p, a rescue experiment was conducted. After circSOD2 knockdown or overexpression, the abundance of miR-532-3p was altered and changes in cell function were observed. Knockdown of circSOD2 reduced the proliferation, migration, invasion, and viability of 786-O cells, but the inhibitory effect could be reversed by miR-532-3p inhibitors (Fig. 8A, B, E). Similar regulation was observed in 769P cells overexpressing circSOD2 and miR-532-3p mimics (Fig. 8C, D, E).

Western blot assays also showed results consistent with functional changes. In 786-O cells, the activity of PAX5 and p-PI3K proteins decreased after transfection with miR-532-3p mimics, and this alteration could be rescued by transfecting the circSOD2 overexpression vector. In contrast, after transfection with miR-532-3p inhibitors, PAX5 and p-PI3K were activated, but this effect was weakened by the introduction of circSOD2 shRNA in 769P cells (Fig. 8F). A diagram of the related signaling pathway is shown in Fig. 8G.

### PAX5 enhances the biosynthesis of circSOD2 in ccRCC cells

**Fig. 9:**
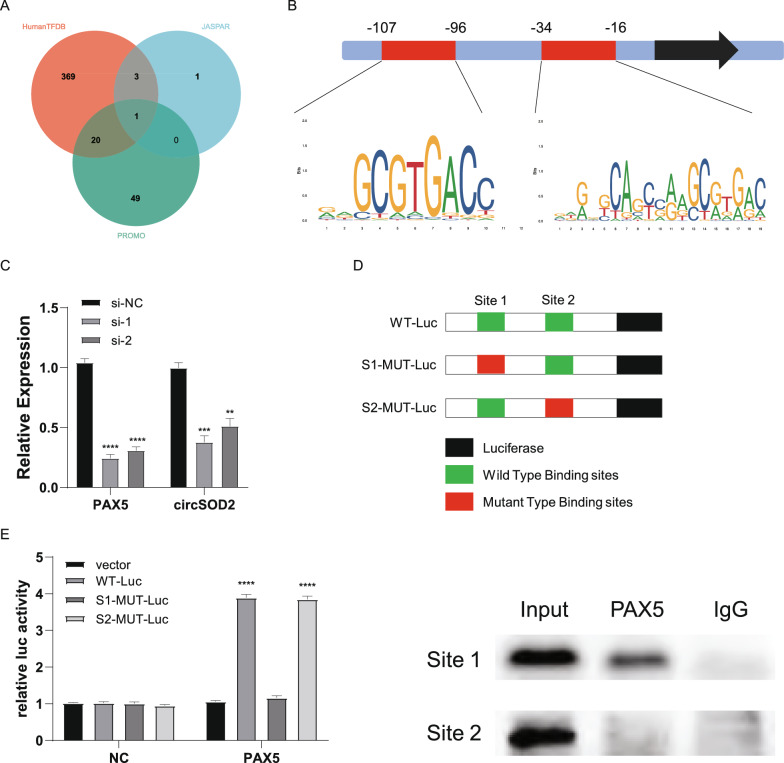
PAX5 enhances the biosynthesis of circSOD2 in ccRCC cells. **A** Venn diagram shows the intersection of transcription factors predicted by the three databases. **B** PAX5 has two possible transcription factor binding sites in the promoter of circSOD2. **C** qRT-PCR showed that the expression of circSOD2 decreased with the knockdown of the expression of PAX5 in 786-O cells. **D** Schematic diagram of luciferase reporter vectors containing mutations at each of these two sites (S1-MUT-Luc and S2-MUT-Luc). **E** Luciferase assays proved the the expression of PAX5 significantly activated the site 1, rather than the site 2. **F** ChIP assay showed that PAX5 was enriched at site 1. (NS, nonsignificant; *P < 0.05; **P < 0.01; ***P < 0.001; ****P < 0.0001)

The production of circRNAs is regulated by a variety of factors [45–47], and PAX5, a transcription factor, can promote the synthesis of various circRNAs [42]. To further explore the transcriptional regulatory mechanism of circSOD2 in ccRCC, we used the JASPAR [48] (https://jaspar.elixir.no/), HumanTFDB [49] (http://bioinfo.life.hust.edu.cn/HumanTFDB#!/), and PROMO [50] (https://alggen.lsi.upc.es/cgibin/promo_v3/promo/promoinit.cgi?dirDB=TF_8.3) databases to search for possible transcription factors that might regulate circSOD2. As the factor with the highest overall score, two possible transcription factor binding sites (TFBS) in the promoter of circSOD2 (site 1: − 107 ~ − 96, *tgcgtgagcac*; site 2: − 34 ~ − 16, *gaggcagcagtgctcagc*) were predicted (Fig. 9A, B). When we knocked down PAX5 in 786-O cells, the expression of circSOD2 was also reduced (Fig. 9C). We then designed vectors containing mutations at each of the two sites for the luciferase reporter assays. The results showed that the expression of PAX5 significantly activated site 1, but not site 2 (Fig. 9D, E). To determine whether PAX5 directly binds to the circSOD2 promoter, ChIP assays were performed and it was found that PAX5 was enriched at site 1 (Fig. 9F). These data suggest that transcription factor of PAX5 accelerates the biogenesis of circSOD2.

## Discussion

Over the past several years, it has been believed that circRNAs, which were discovered several decades ago [8, 51], are produced as a consequence of incorrect splicing during transcription in eukaryotes [52]. With the development of bioinformatics and high-throughput sequencing, the biological functions of an increasing number of circRNAs have been discovered and explored [10]. In this context, human cells have more unique circRNAs than protein-coding genes [53]. Abundant studies have proven that circRNAs play a considerable role in the development and progression of various malignant diseases, and either the extensive existence or regulatory mechanisms have made circRNAs certified biomarkers and therapeutic targets [54–60]. Nevertheless, the functions of circRNAs in the progression of ccRCC remain unclear.

Here, we identified a novel circRNA, circSOD2, that was significantly differentially expressed in two microarray datasets from ccRCC patients. Subsequent studies demonstrated that circSOD2 was upregulated in ccRCC cell lines and samples compared to normal cells and tissues. The expression level of circSOD2 showed a positive correlation with both the TNM stage and Fuhrman grade of ccRCC patients, and higher levels of circSOD2 were associated with poorer survival outcomes, including OS and DFS. Furthermore, a series of in vitro and in vivo experiments proved that the reduction of circSOD2 suppressed proliferation, migration, and invasion, and stimulated apoptosis and programmed cell death of ccRCC cells, whereas the overexpression of circSOD2 had the opposite effect.

Increasing evidence supports that circRNAs serve as miRNA sponges and affect downstream target genes to mediate various biological functions [15, 53, 61]. For instance, circRIP2, a competing endogenous RNA (ceRNA), binds to miR-1305 in cells and promotes the progression of bladder cancer [62]. In this study, we showed that circSOD2 was predominantly present in the cytoplasm of ccRCC cells. Therefore, we hypothesized that circSOD2 might act as a miRNA sponge to influence ccRCC progression. Following high-throughput sequencing of miRNA, luciferase analysis, and miRNA pull-down analysis, we confirmed that there is a close interaction between circSOD2 and miR-532-3p. Studies have indicated that miR-532-3p can suppress the progression of several types of tumors, such as lymphoma [63], colorectal cancer [64] and prostate cancer [65]. The two patient cohorts used in our study showed the same phenomenon. A negative correlation was observed between circSOD2 and miR-532-3p expression levels, and the prognosis of ccRCC patients was worse in cases where miR-532-3p levels were lower. Consistent with this, in vitro experiments also demonstrated the antitumor effect of miR-532-3p. In addition, the overexpression of circSOD2 attenuated the inhibition of miR-532-3p in ccRCC. These results indicated that circSOD2 interacts with miR-532-3p to perform specific cellular functions.

Increasing evidence suggests that circRNAs play a key role regarding the metastasis of genitourinary cancers. Additionally, circRNAs have been proposed as prospective biomarkers and therapeutic targets for cancer, with urologic cancer included, for its stability and specific expression. Since the spread of cancer cells from the primary tumor site to distant tissues involves a series of sophisticate steps [66, 67], circRNAs exert their pivotal roles in epithelial-mesenchymal transition (EMT), local invasion and migration, endocytosis by breaching the vascular wall to enter blood and lymphatic vessels, exocytosis in the vessels by recruitment of macrophages metastatic colonization, and regulation of the immune microenvironment. Recently, with advances in bioinformatics and high-throughput sequencing technologies have led to the identification of numerous circRNAs that are known to exhibit important roles in cancer, including ccRCC [68]. Shen et al. demonstrated that circATG9A played a crucial role in promotion of EMT process through the miR-497-5p/TRPM3/Wnt/β-catenin axis [69]. With the use of enrichment analysis and correlation analysis of high-through RNA sequencing data, Cen et al. identified has_circ_0057105 as a potential oncogenic regulator targeted to COL1A1 in the activation of EMT in RCC [70], while it was also found to be involved in the process of ferroptosis regulation through target gene VDAC2. Such dual regulatory roles regarding a specific circRNA may provide new insights into our understanding of the balance between EMT and ferroptosis in RCC. Besides the demonstration of inhibitory effect of circNTNG1 in RCC aggression, Liang et al. identified the circNTNG1/miR-19b-3p/HOXA5 axis can exert regulatory effects on epigenetic silencing of Slug, resulting in EMT interference and metastasis of RCC [71]. Another regulatory axis, circESRP1/miR-3942/CTCF, was revealed by Gong et al., and they validated that CTCF can precisely promote circESRP1 transcript expression, thus creating a positive-feedback loop in ccRCC progression [72]. Apart from the already mentioned positive feedback loop, the circNIPBL/miR-16-2-3p/Wnt5a/ZEB1 axis in bladder cancer [73], circMGA/HNRNPL complex as a pivotal tumor suppressor by recruiting CD8 + T cells in bladder cancer [74], and loop among circCDK13/miR-212-5p/449a/E2F5 in prostate carcinogenesis [75] were also shown to participate in tumor progression. Our study identified circSOD2 with significant tumor-promoting effects in RCC based on bioinformatics and preliminary validation of clinical samples. Subsequent sequencing analyses, phenotypic explorations and mechanistic experiments further confirmed that circSOD2 promoted RCC metastasis through the circSOD2/miR-532-3p/PAX5 positive feedback pathway. According to our knowledge, circSOD2 have been shown to be significantly decreased in the urine of ccRCC patients [17]. Although the reason for the different expression profiles between urine and tumor tissues is not cleat, it provides ideas for future exploration of the reasons for the changes in circRNA expression levels in both urine and tumor samples of patients with ccRCC, and whether circSOD2 could be a promising marker for liquid biopsy for diagnosis and risk stratification. In addition, given the high risk of metastasis of ccRCC, circSOD2 was shown to contribute to EMT-related progression in non-small cell lung cancer [35], indicating that it may serve as a metastatic predictive marker in ccRCC.

Along with exploring biomarkers that may have an impact on EMT, invasion or metastasis, the advancement of precision medicine has been fueled by the emergence of immunotherapies, including immune checkpoint inhibitors (ICIs) and messenger RNA vaccines [76, 77]. Ye et al. found that circSOD2 was related to anti-PD-1 resistance in hepatocellular carcinoma (HCC) throught miR-297-5p/ANXA11 pathway [78]. In specific, they discovered that high expression of circSOD2 would impair CD8 + T cell viability and thus facilitates immune evasion in HCC. In gastric cancer, Qu et al. found that circSOD2 would enhance M1 polarization of macrophages to alleviate the cisplatin resistance through miR-1296/STAT1 axis [79]. Taken together, perhaps it would be instructive to target the immunomodulatory role of circSOD2 in RCC to explore its potential value in complementing existing immunotherapies.

Besides, it is commonly accepted that miRNAs can weaken the expression level of target genes by binding to sites within the 3’ untranslated regions (3’ UTR) of mRNAs [80–83]. In this study, PAX5 was identified as a direct target of miR-532-3p by bioinformatics analysis and later confirmed by dual-luciferase reporter assays. In addition to the experiments which verified PAX5 has a carcinogenic effect in ccRCC cells, further rescue experiments revealed that circSOD2 inhibited the expression of miR-532-3p, thereby alleviating the inhibitory effect of miR-532-3p on PAX5.

The biosynthesis of circRNAs involves multiple factors, including the transcription of circRNA precursor and back-splicing, in which transcription factors play an important role. For example, c-Myc, in combination with splicing factor 10 (SRSF10), can promote the transcription and back-splicing of CAMSAP1 precursor mRNA, leading to the proliferation and metastasis of nasopharyngeal carcinoma [84]. E2F5 upregulation enhances CDK13 transcription and promotes circCDK13 biosynthesis, leading to prostate carcinogenesis [75]. PAX5, functioning as a classical transcription factor, can regulate the expression of many genes, such as MYC [85, 86], WAPL [44], and several type 2 diabetes-related genes, including SFRP1 and SYT12 [87]. In our study, we demonstrated that PAX5 can promote the synthesis of circSOD2 and thus promote the development of RCC, which further supports that circSOD2 may be a biomarker for risk stratification and prognostic prediction in ccRCC patients. However, the precise mechanism by which PAX5 affects circSOD2 requires further investigation. Overall, this study provides a novel closed-loop structure for the potential pathogenic mechanisms of ccRCC.

## Conclusions

In conclusion, we demonstrated that circSOD2 acts as an oncogenic agent that promotes ccRCC proliferation and metastasis through the formation of the PAX5/ circSOD2/miR-532-3p/ PAX5 loop. In addition to elucidating the mechanism through which circSOD2 controls the progression of ccRCC, this discovery also presents a promising new biomarker and therapeutic target for the clinical management of ccRCC patients.

### Supplementary Information

Supplementary Material 1.

## Data Availability

The datasets used in this study are accessible through the Cancer Genome Atlas (https://portal.gdc.cancer.gov/) and GEO (http://www.ncbi.nlm.nih.gov/geo/) with accession numbers GSE100186 and GSE137836.

## Abbreviations

ccRCC: Clear cell renal cell carcinoma
GEO: Gene Expression Ominibus
qRT-PCR: Quantitative real-time PCR
OS: Overall survival
circRNA: Circular RNA
PAX5: Paired box 5
SSIGN: Stage, dimension, grade, and necrosis
shRNA: Short hairpin RNA
siRNA: Small interfering RNA
ROC: Receiver operating characteristic
