# Supplementary material for: Exploring the oncogenic potential of circSOD2 in clear cell renal cell carcinoma: a novel positive feedback loop
Source: J Transl Med. 2024 Jun 27;22:596. doi: 10.1186/s12967-024-05290-9 (PMC11209967; doi:10.1186/s12967-024-05290-9)
Supplement: Supplementary file 1 — Supplementary Material 1. [file 12967_2024_5290_MOESM1_ESM.docx]

Supplementary Materials


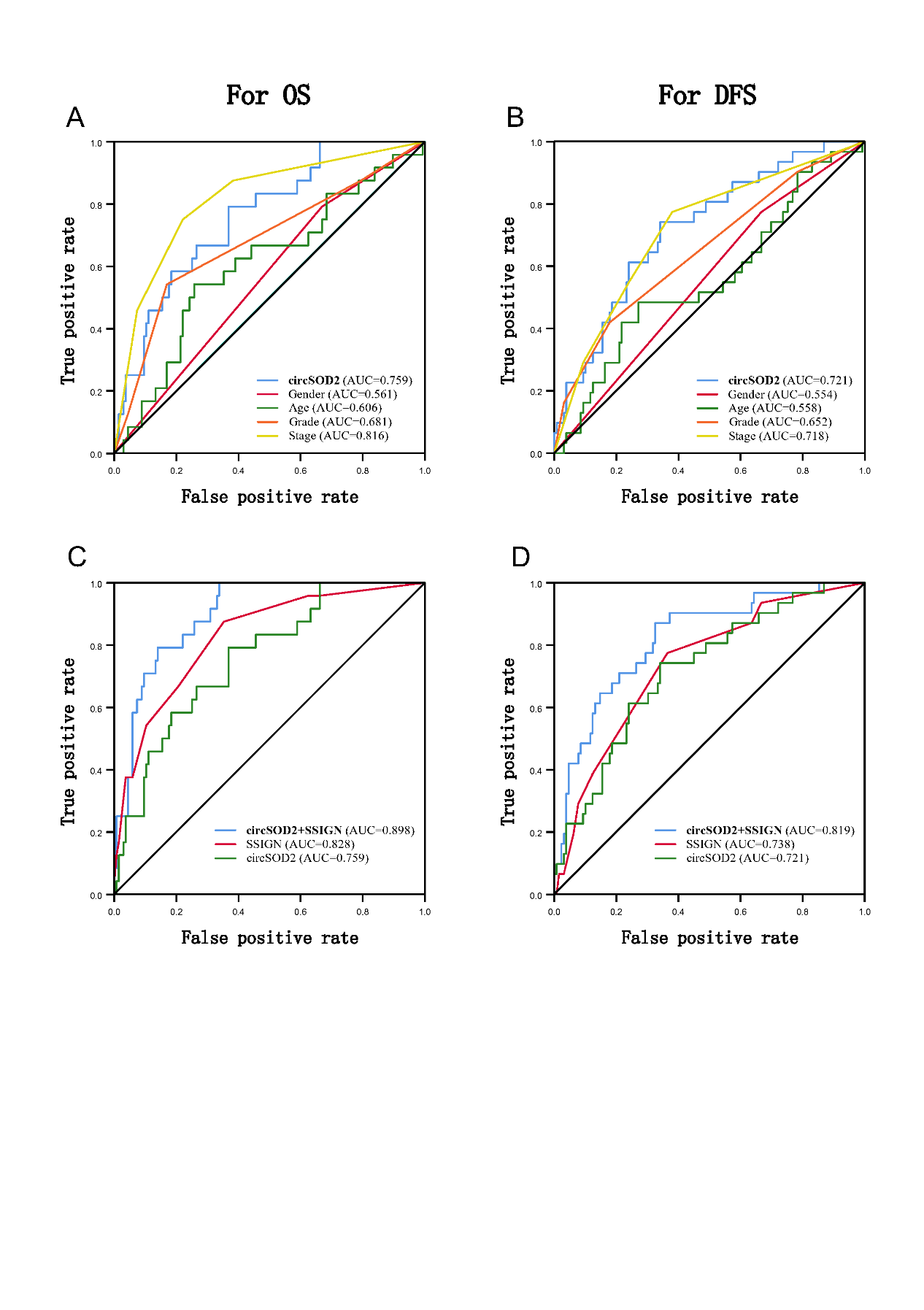


**Figure S1.** circSOD2 expression level combined with different clinical parameters of receiver operating characteristic (ROC) analysis.


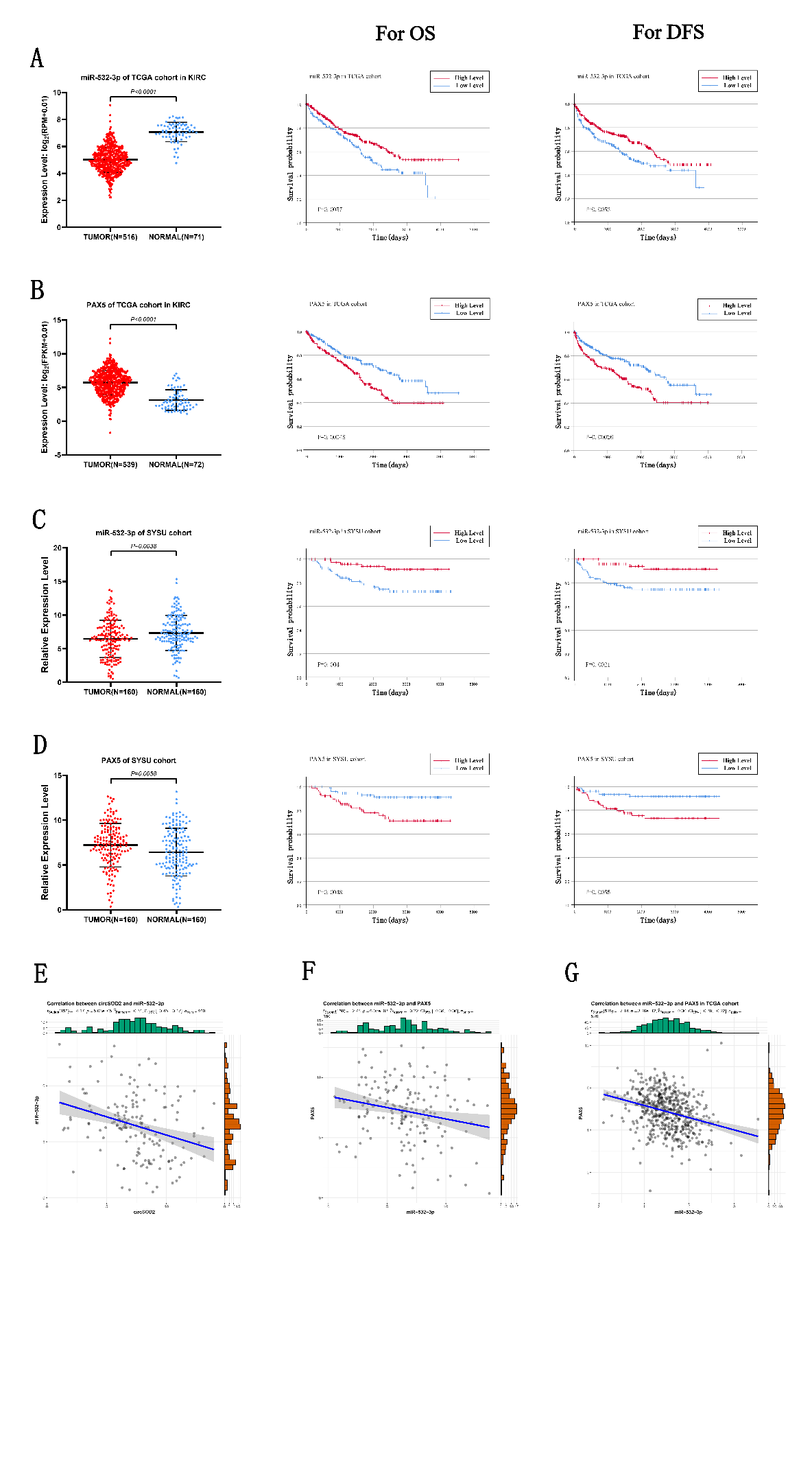


**Figure S2.** **A,** Expression difference and Kaplan-Meier curve of miR-532-3p in tumor and normal tissues in TCGA cohort. **B,** Expression difference and Kaplan-Meier curve of PAX5 in tumor and normal tissues in TCGA cohort. **C,** Expression difference and Kaplan-Meier curve of miR-532-3p in tumor and normal tissues in SYSU cohort. **D,** Expression difference and Kaplan-Meier curve of PAX5 in tumor and normal tissues in SYSU cohort. **E,** The expression level of miR-532-3p was negatively correlated with that of circSOD2 in SYSU cohort. **F,** The expression level of PAX5 was negatively correlated with that of miR-532-3p in SYSU cohort. **G,** The expression level of PAX5 was negatively correlated with that of miR-532-3p in TCGA cohort.

| **Table S1. The primers and related sequences used in this study** | |
| --- | --- |
| **Substances** | **Sequences (5′-3′)** |
| **Primers** |  |
| GAPDH Forward | GGAGCGAGATCCCTCCAAAAT |
| GAPDH Reverse | GGCTGTTGTCATACTTCTCATGG |
| U6 Forward | CTCGCTTCGGCAGCACA |
| U6 Reverse | AACGCTTCACGAATTTGCGT |
| circSOD2 Forward | CTGCTGGGGATTGATGTGTG |
| circSOD2 Reverse | TCTGGGCTGTAACATCTCTCA |
| SOD2 Forward | GGAAGCCATCAAACGTGACTT |
| SOD2 Reverse | CCCGTTCCTTATTGAAACCAAGC |
| PAX5 Forward | ACAGCATAGTGTCCACTGGC |
| PAX5 Reverse | ATAGTAGGGGGAGCCAAGCA |
| Universal RT primer | CAGTGCAGGGTCCGAGGTCAGAGCCACCTGGGCAATTTTTTTTTTTVN |
| Universal reverse primer | CAGTGCAGGGTCCGAGGT |
| miR-532-3p Forward | AACAGTGCATGCCTTGAGTGTAG |
| miR-507 Forward | AAGCGCCTTTTTGCACCTTTTG |
| miR-188-3p Forward | AACAAGCTCCCACATGCAGG |
| miR-135a-5p Forward | CGCTGGTATGGCTTTTTATTCCT |
| miR-129-5p Forward | AACCACTCTTTTTGCGGTCTGG |
| **siRNAs and shRNAs** |  |
| sh-NC | GCGACGATCTGCCTAAGAT |
| circSOD2-sh1 | ATCGTTATGCTGAGAGATGTT |
| circSOD2-sh2 | TATGCTGAGAGATGTTACA |
| PAX5-si1 | GGTAATTGGAGGATCCAAACC |
| PAX5-si2 | GCCGACACCAACAAGCGCAAG |
| **miRNA mimics**  **and inhibitors** |  |
| mimics NC | AUUGGAACGAUACAGAGAAGAUU |
| miR-532-3p mimics | CCUCCCACACCCAAGGCUUGCA |
| inhibitor NC | CAGUACUUUUGUGUAGUACAA |
| miR-532-3p inhibitor | UGCAAGCCUUGGGUGUGGGAGG |

| **Table S2. Most significantly different circRNAs in the two GEO datasets (\|logFoldChange\|>3).** | | | | | |
| --- | --- | --- | --- | --- | --- |
| **circular RNAs** | **Position:**  **(Start-End)** | **Gene symbol** | **logFC in**  **GSE100186** | | **logFC in**  **GSE137836** |
| **hsa_circ_0000317** | *Chr11:62288378-62288522* | **AHNAK** | | 3.3439046 | 4.048782 |
| **hsa_circ_0000319** | *Chr11:62291306-62292503* | **AHNAK** | | 3.7206668 | 4.790296 |
| **hsa_circ_0000740** | *Chr17:5364258-5365866* | **DHX33** | | 3.7229334 | 5.848821 |
| **hsa_circ_0000741** | *Chr17:7402357-7402810* | **POLR2A** | | 3.7649842 | 3.184173 |
| **hsa_circ_0001873** | *Chr9:93637042-93639999* | **SYK** | | 4.6663986 | 6.102336 |
| **hsa_circ_0001946** | *ChrX:139865339-139866824* | **CDR1** | | 3.4617229 | 4.898622 |
| **hsa_circ_0001952** | *ChrX:154018228-154020560* | **MPP1** | | 4.2325006 | 3.65301 |
| **hsa_circ_0001968** | *Chr11:68359043-68367962* | **PPP6R3** | | 4.8930035 | 4.901037 |
| **hsa_circ_0002211** | *Chr22:38890634-38897285* | **DDX17** | | 3.1182713 | 3.194711 |
| **hsa_circ_0003596** | *Chr9:137716445-137717750* | **COL5A1** | | 4.7398897 | 4.718555 |
| **hsa_circ_0004163** | *Chr18:662145-671451* | **TYMS** | | 3.6536947 | 3.580047 |
| **hsa_circ_0004662** | *Chr6:160103505-160109274* | **SOD2** | | 4.2205186 | 3.101044 |
| **hsa_circ_0006050** | *Chr8:133740043-133769540* | **TMEM71** | | 3.9161368 | 3.704028 |
| **hsa_circ_0006528** | *Chr5:145197456-145205763* | **PRELID2** | | 3.7158499 | 3.020911 |
| **hsa_circ_0006562** | *Chr7:65915057-66041919* | **TCONS_l2_00027391** | | 4.4157701 | 5.356012 |
| **hsa_circ_0015004** | *Chr1:161293403-161326630* | **SDHC** | | 3.4643335 | 3.115237 |
| **hsa_circ_0020303** | *Chr10:125771848-125806240* | **CHST15** | | 4.4684843 | 6.085663 |
| **hsa_circ_0030431** | *Chr13:75936161-75936743* | **TBC1D4** | | 4.4889315 | 5.02296 |
| **hsa_circ_0031594** | *Chr14:34398281-34400421* | **EGLN3** | | 5.3203747 | 3.21774 |
| **hsa_circ_0037866** | *Chr16:11778020-11830089* | **TXNDC11** | | 4.6093964 | 4.808629 |
| **hsa_circ_0039353** | *Chr16:53189837-53191453* | **CHD9** | | 3.2019955 | 3.146021 |
| **hsa_circ_0047463** | *Chr18:34376033-34378572* | **TPGS2** | | 4.5403039 | 4.83886 |
| **hsa_circ_0058792** | *Chr2:236617822-236659132* | **AGAP1** | | 4.4208797 | 4.733474 |
| **hsa_circ_0058794** | *Chr2:236626200-236659132* | **AGAP1** | | 4.6681675 | 4.862766 |
| **hsa_circ_0065217** | *Chr3:47468646-47470160* | **SCAP** | | 3.6188947 | 3.873663 |
| **hsa_circ_0092338** | *Chr20:25477659-25477979* | **NINL** | | 4.2229444 | 4.411539 |

| **Table S3. The most statistically significant candidate miRNAs.** | | | | | |
| --- | --- | --- | --- | --- | --- |
| **miRNA** | **log2(sh/NC)** | **log2FoldChange** | **pvalue** | **qvalue** | **result** |
| *hsa-miR-106b-3p* | 8.166684549 | 7.344325528 | 2.67E-224 | 3.71E-222 | up |
| *hsa-miR-125b-5p* | 9.426510263 | 8.548987522 | 1.44E-18 | 1.47E-17 | up |
| *hsa-miR-132-3p* | 5.992641977 | 5.038653611 | 1.14E-21 | 1.32E-20 | up |
| *hsa-miR-188-3p* | 8.936637939 | 8.153445993 | 5.88E-15 | 5.28E-14 | up |
| *hsa-miR-26a-1-3p* | 15.75824924 | 7.138600736 | 9.41E-11 | 6.93E-10 | up |
| *hsa-miR-342-3p* | 6.358665915 | 5.480683772 | 3.77E-152 | 3.58E-150 | up |
| *hsa-miR-345-5p* | 7.660149414 | 6.794555139 | 9.37E-58 | 3.19E-56 | up |
| *hsa-miR-362-5p* | 3.852669253 | 2.975658027 | 1.48E-109 | 8.88E-108 | up |
| *hsa-miR-532-3p* | 15.42908771 | 14.65484279 | 6.88E-34 | 1.38E-32 | up |
